# Fumonisin B_1_ Toxicity in Grower-Finisher Pigs: A Comparative Analysis of Genetically Engineered Bt Corn and non-Bt Corn by Using Quantitative Dietary Exposure Assessment Modeling

**DOI:** 10.3390/ijerph8083179

**Published:** 2011-07-28

**Authors:** James E. Delgado, Jeffrey D. Wolt

**Affiliations:** Interdepartmental Toxicology Program, Department of Agronomy, Iowa State University, Ames, IA 50011, USA; E-Mail: jdwolt@iastate.edu

**Keywords:** *Bacillius thuringiensis corn*, Bt corn, swine diet, DDGS, fumonisin, risk assessment

## Abstract

In this study, we investigate the long-term exposure (20 weeks) to fumonisin B_1_ (FB_1_) in grower-finisher pigs by conducting a quantitative exposure assessment (QEA). Our analytical approach involved both deterministic and semi-stochastic modeling for dietary comparative analyses of FB_1_ exposures originating from genetically engineered *Bacillus thuringiensis* (Bt)-corn, conventional non-Bt corn and distiller’s dried grains with solubles (DDGS) derived from Bt and/or non-Bt corn. Results from both deterministic and semi-stochastic demonstrated a distinct difference of FB_1_ toxicity in feed between Bt corn and non-Bt corn. Semi-stochastic results predicted the lowest FB_1_ exposure for Bt grain with a mean of 1.5 mg FB_1_/kg diet and the highest FB_1_ exposure for a diet consisting of non-Bt grain and non-Bt DDGS with a mean of 7.87 mg FB_1_/kg diet; the chronic toxicological incipient level of concern is 1.0 mg of FB_1_/kg of diet. Deterministic results closely mirrored but tended to slightly under predict the mean result for the semi-stochastic analysis. This novel comparative QEA model reveals that diet scenarios where the source of grain is derived from Bt corn presents less potential to induce FB_1_ toxicity than diets containing non-Bt corn.

## 1. Introduction

Fumonisins are a series of mycotoxins ubiquitous in Nature, infecting corn (*Zea mays* L*)* and other grains throughout the World. Major fumonisin fungi species-mycotoxin associations are derived from *Fusarium verticilliodes* (formerly known as *F. moniliforme*) and *F. proliferatum.* Minor fumonisin sources include *Fusarium nygamai, F. napiforme*, *F. thapsinum*, *F. anthophilum and F. dlamini* [1]. Detection of mycotoxicosis usually involves a close association between the consumption of moldy feed and a specific onset of toxicological effects, altered performance or behavior. Fumonisin-induced porcine pulmonary edema (PPE) is a well-established toxin specific adverse effect [2], and fumonisin also has the potential to negatively impact the food and feed market due to contaminated grain [3].

We recently reported after conducting an exposure assessment that swine populations in nursery facilities may frequently exhibit incipient fumonisin B_1_ (FB_1_) toxicological effects (*i.e.*, 8% decrease in average daily weight gain) when diets are contaminated at 1 mg of FB_1_/kg of diet. The results of Delgado and Wolt [4] have been largely validated by the recent study of Rossi *et al.* [5] which reports better performance in weaned piglets fed Bt corn compared to piglets fed near isogenic corn and suggests better performance due to lower FB_1_ associated with Bt corn [4,5]. The authors’ goals in this investigation are to better understand the lifetime exposure (*utero*-to-finish) and toxicity of FB_1_ in pig diets. Due to the variation of percent corn in the diet design throughout the lifetime production, we have divided our quantitative exposure assessment (QEA) modeling into three major components: gestation, nursery, and grower-finisher. This investigation is currently focused on the grower-finisher component and will use our previously established analytical exposure model framework. The only variation in the grower-finisher model compared to our previous nursery model is the current inputs reflect diet formulation for grower-finisher pigs.

Quantitative exposure assessment was conducted using both deterministic (single-point estimates) and stochastic (probabilistic) analysis for comparative interpretation of FB_1_ exposure originating from genetically engineered *Bacillus thuringiensis* (Bt)-corn, conventional non-Bt corn and distiller’s dried grains with solubles (DDGS). Comparative analysis between Bt corn and non-Bt corn is conducted to determine if FB_1_ concentrations differ depending on the corn source, estimating which swine populations may be more susceptible to FB_1_ toxicity.

## 2. Materials and Methods

Animal Care and Use Committee approval was not obtained for this study because forecast data were derived from existing literature.

### 2.1. Analytical Model

Characterization of risk from FB_1_ dietary exposure was estimated by using a conceptual model, which consists of three major components: toxicological effects (levels of concern, LOC), swine management, and agronomic management as described in Delgado and Wolt [4]. Six scenarios were developed to consider FB_1_ exposure influenced by corn and DDGS as the primary protein source in diets:

- Scenario 1: Blended diet (Bt grain, non-Bt grain, Bt-DDGS and non-Bt DDGS)
- Scenario 2: Bt grain and Bt DDGS
- Scenario 3: non-Bt grain and non-Bt DDGS
- Scenario 4: Bt and non-Bt grain
- Scenario 5: Bt grain
- Scenario 6: non-Bt grain

### 2.2. Exposure Characterization and Model Parameterization

Information necessary to forecast FB_1_ exposure and model parameterization needed to estimate risk consistent with the conceptual model is presented in the following subsections. Each diet scenario required separate sets of worksheets (Microsoft Excel 2010) to describe the FB_1_ exposure. Deterministic inputs (Table 1) used average, maximum, midpoint or fixed parameter estimates and all probabilistic modeling (Table 1) used Palisade @Risk 5.7 with random Latin hypercube sampling [6]. The term semi-stochastic will be used to refer to the non-deterministic modeling which does not contain distributions for the inputs of specific week in grower-finisher phase, Bt use fraction in diets and estimations of FB_1_ in corn. Refer to Table 1 for descriptions of model input assumptions.

*Swine Management*. Model parameterization required for diet development included the following: mycotoxin exposure assessed by weekly intervals during the production phase, changes in body weight (BW) over time (*i.e.*, weekly), and total corn intake fraction (TCIF). Information for modeling the diet reflected a typical corn-soybean diet for swine facilities in the Midwestern USA.

*Duration of Exposure (Weekly).* For the purpose of this dietary exposure assessment, weekly intervals were modeled in order to estimate variations of FB_1_ in diets. Estimating exposure by daily intervals was not conducted due to limited changes in diet composition. The sampling of the weekly intervals (*i.e.*, 20 weeks) during production allows for an estimated correlated BW and expected TCIF in accordance with the Kansas State University Growth and Feed Intake Curve Calculator (FICC, see BW and TCIF below). All deterministic modeling scenarios used the 10^th^ week of production to represent the midpoint of duration. For the semi-stochastic analysis a total of 20 weekly intervals of production were partitioned into six timeframes representative of weight ranges corresponding to the TCIF (Table 2 and Table 3) and sampled by a discrete uniform distribution to estimate the body weight associated with weekly interval.

*Bodyweight (BW)*. Determination of BW was calculated by the Kansas State University Growth FICC as a function of the specific week during production [7]. Parameterization inputs for the FICC included initial nursery average BW of 5.67 kg and an average daily gain of 0.39 kg. Initial BW of grower-finisher production was 22.68 kg with an average daily gain of 0.82 kg, and 120.20 kg as the close out average BW. Values of BW were calculated at the end of the indicated week after placement into the grower-finisher phase (Table 2).

*Total Corn Intake Fraction.* Estimation of the TCIF in diet is based on the BW intervals associated within the 20 week production duration (Table 3) [9].

### 2.3. Agronomic Management

*Bt vs. non-Bt Corn Fraction in Diet.* Estimation of the fraction of Bt and non-Bt corn in swine diets was conducted by using the percentage of US hectares planting Bt and non-Bt seed corn. The USDA National Agricultural Statistics Service (NASS) estimated in 2010 that 15% of corn planted in the state of Iowa was insect-resistant (Bt) and 61% of all corn planted in Iowa was stacked gene varieties (Bt plus herbicide resistance) [10]. Therefore, in our deterministic model we assume that the TCIF in swine diets has a maximum Bt use fraction (BUF) representing 76% of Iowa corn planted, whereas the stochastic analysis distribution was developed from hectares planted in the major corn production states of the US [10]. For stochastic analysis Bt-corn adoption fractions were estimated by using a beta generalized distribution as described by Delgado and Wolt (Table 4) [4].

*DDGS Fraction in Diet*. In the Midwestern USA DDGS is increasingly used as an alternative feed source due to increased prices of corn and the widespread availability of DDGS as a by-product of ethanol production. Producers usually design the diets to use the maximum allowed percentage of DDGS. Therefore, DDGS distributions were not used in the models. Both deterministic and semi-stochastic modeling used a maximum of 30% DDGS in the diet formulation, since this value represents acceptable growth performance for swine in the grower-finisher phase [8].

**Figure 1.**
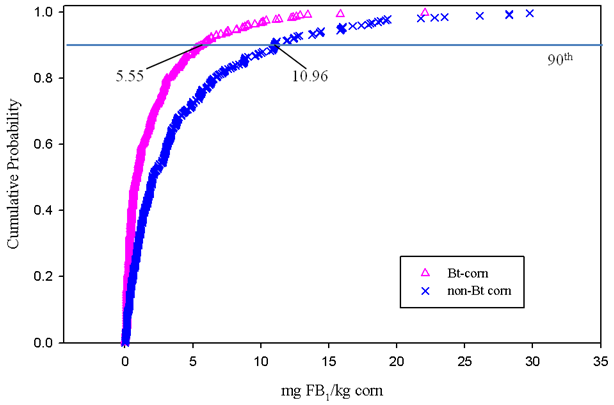
Cumulative distribution of fumonisin B_1_ (FB_1_) concentrations (mg of FB_1_/kg corn) in Bt (*Bacillius thuringiensis*) *vs.* non-Bt corn; data from 1999 to 2006 [11–21] from Delgado and Wolt [4].

*Fumonisin B_1_ Concentrations in Bt-hybrids, Non-Bt Hybrids, and DDGS*. Paired trials of Bt and non-Bt hybrids were used for estimates of FB_1_ in diets, which were expressed as cumulative distribution functions (CDF) describing the empirical data (Figure 1) [11–21]. For specific details pertaining to the CDF calculations, see Delgado and Wolt [4]. Estimates of FB_1_ concentration in DDGS used a 3-fold scaling for both deterministic and semi-stochastic analysis as a typically reported value [3].

**Figure 2.**
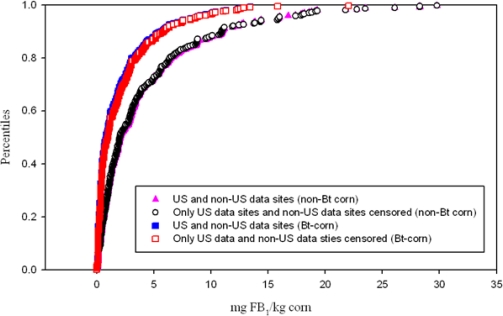
Comparison of US and non-US data *versus* censoring non-US data showing a cumulative distribution of fumonisin B_1_ (FB_1_) concentrations (mg of FB_1_/kg corn).

Information used to generate CDF contains both US and non-US data. We considered very carefully the source data and rationale for inclusion of non-US data sites. Rationale for inclusiveness is to better represent the potential variation in FB_1_ due to diverse genetic backgrounds and environments (e.g., location and years). The inclusion of non-US data represents 8.31% (*i.e.*, 32 observations in a total of 385) of the total data used to represent FB_1_ in corn (Figure 2).

### 2.4. Effects Characterization

Chronic toxicological adverse effects associated with FB_1_ concentrations relevant to dietary exposure in the grower-finisher production phase for formulating the incipient level of concern (LOC) are reviewed in depth by Delgado and Wolt [4] and include the toxicological study of Rotter *et al.* [22]. The LOC for this QEA is 1.0 mg of FB_1_/kg of diet, which is consistent with the lower LOC used by Delgado and Wolt in the QEA for swine in nurseries [4].

## 3. Results

### 3.1. Deterministic Results

Existing data were used to forecast long-term FB_1_ exposures in feeding scenarios which may occur in the swine industry. Risk findings were expressed as the probability for exposures to exceed the LOC for long-term effects (1 mg FB_1_/kg diet). All diet scenarios predicted some level of FB_1_ exposure exceeding the LOC (Table 5). Diet scenarios where the source of grain or DDGS is derived from non-Bt corn (scenarios 3 and 6) pose the greatest opportunity for exceeding the LOC. Scenarios including only Bt grain (scenario 5) without DDGS exhibited the least mycotoxin exposure. The blended diet design (scenario 1) containing Bt and non-Bt grain and DDGS was ranked intermediate relative to other diet scenarios.

### 3.2. Semi-Stochastic Results

**Figure 3.**
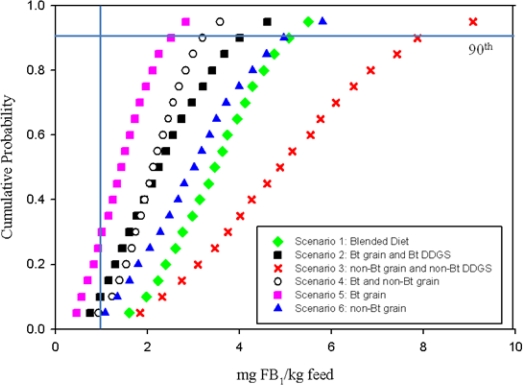
Cumulative distributions of chronic fumonisin B_1_ (FB_1_) exposure in grower-finisher pig diet scenarios compared to the lower threshold of concern (1 mg FB_1_/kg diet). Blended diet contains Bt grain, non-Bt grain, Bt DDGS, non-Bt DDGS.

FB_1_ exposures exceeding the LOC were forecasted for all diet scenarios (Figure 3). Variation of FB_1_ exposure among scenarios and worst-case incidences representing the 90th percentile of exposure (Table 5) showed the least risk when the diets were developed with Bt grain only (scenario 5) while non-Bt and non-Bt DDGS diets (scenario 3) showed the highest LOC exceedance in 95% of cases. The percentile exceedance of LOC (1 mg FB_1_/kg diet) forecasted were:

- Scenario 1: Blended diet (95% of occasions)
- Scenario 2: Bt-grain and Bt DDGS (85% of occasions)
- Scenario 3: non-Bt and non-Bt DDGS (95% of occasions)
- Scenario 4: Bt-grain and non-Bt grain (90% of occasions)
- Scenario 5: Bt grain (70% of occasions)
- Scenario 6: non-Bt grain (95% of occasions)

## 4. Discussion

Semi-stochastic results predicted FB_1_ ranging from 1.50 to 5.08 and 2.52 to 7.87 mg FB_1_/kg diet for the mean and 90th percentile, respectively, where the chronic toxicological incipient level of concern is 1.0 mg of FB_1_/kg of diet. Due to the lack of toxicological data in grower-finisher pigs, it is difficult to predict the possible adverse effects induced above the LOC. Additional studies will be required to fully understand the potential negative impact(s) that may be generated from chronic low-dose exposure to FB_1_ diets. It is worth noting that the blended diet (scenario 1) may represent the swine industry as a whole; however, it is more likely that diets will contain 1 type of corn source or 1 type of DDGS. Methods of preventing, decontaminating and minimizing the toxicity of mycotoxins in feeds has been discussed by Jouany [23].

Long-term, low-dose exposures to FB_1_ in swine feed (as well as in the diets for other sensitive species with a large component of corn and/or DDGS) may represent a factor limiting health and productivity even when FB_1_ is controlled to levels below the acute advisory limits. Both our previous QEA and the recent study of Rossi *et al*. show any potential concern for FB_1_ chronic toxicity in nursery production will be largely alleviated by the use of Bt corn in the feed [4,5]. In order to understand the lifetime exposure (*utero*-to-finish) of FB_1_, further QEA models will be required for the gestation phase. This novel Bt and non-Bt comparative dietary QEA model may assist researchers in the dosimetry exposure characterization of experimental designs.

### Uncertainties in Assessment

Our current model did not include environmental factors inputs, such as temperature, insect pressure, and storage practice variations [24]. However, since we have used data for FB_1_ corn spanning multiple use environments and seven growing seasons, the effects of environmental factors is represented in our sampling distribution.

Estimating the DDGS concentration factor of a 3-fold increase is an overestimate of FB_1_ in diets. Preliminary research to determine the DDGS FB_1_ concentration factors is estimated to range from 1.5 to 2.8 fold [25]. Inclusion of 30% DDGS throughout the entire grower-finisher production phase has been documented to induce softer pork fat due to high concentrations of linoleic acid in the oil of DDGS, resulting in pork fat iodine that are not acceptable. Therefore, recommendations suggest the removal of DDGS at least 3 weeks before slaughter [8]. The current model included DDGS in diets throughout the production phase without removal.

## Figures and Tables

**Table 1.** Scenario 1 deterministic (single-point estimate) and semi-stochastic (probabilistic) analysis input assumptions for estimating long-term (20 weeks) exposure to fumonisin B_1_ in grower-finisher pig diets^1^.

**Input Parameter**	**Deterministic**		**Semi-stochastic**	
	**Value**	**Rationale**	**Distribution**	**Parameters**
**Specific Week in Grower-Finisher**			Discrete	range: 1 to 20
**Phase, (week, wk)^2^**	10.00	midpoint	Uniform	
**Body Weight^2^, kg**	79.4	FICC^2^	BW = *f*(wk)	FICC^2^
**Bt Use Fraction, (BUF)^3^**	0.76	maximum	Generalized	min = 0.47
			Beta^4^	max = 0.69
				mean = 0.57
				mode = 0.49
				*p* = 1.02
				*q* = 1.23
**DDGS Use Fraction, (DUF)^5^**	0.30	maximum	maximum	
**Total corn intake fraction (TCIF), kg corn/kg diet^6^**	0.820	TCIF=*f*(BW)	TCIF = *f*(BW)	
**Fumonisin B_1_ concentration in Bt grain, mg FB_1_/kg corn, ([FB_1_]Bt)**	2.05	arithmetic mean	empirical CDF^7^	min = 0.01
				1% = 0.02
				5% = 0.11
				10% = 0.14
				25% = 0.28
				50% = 0.85
				75% = 2.69
				90% = 5.59
				95% = 8.22
				99% = 13.43
				max = 22.50
**Fumonisin B_1_ concentration in non-Bt grain, mg FB_1_/kg corn, ([FB_1_]non-Bt)**	4.15	arithmetic mean	empirical CDF^7^	min = 0.00
				1% = 0.05
				5% = 0.14
				10% = 0.28
				25% = 0.78
				50% = 2.05
				75% = 5.59
				90% = 11.03
				95% = 15.91
				99% = 28.28
				max = 54.45
**DDGS Concentration Factor (DCF)^8^**	3.00	fixed	fixed	

1 Fumonisin B_1_ exposure equation: TCIF × [FB_1_]Bt [(BUF – DUF) + (DUF × DCF)] + TCIF × [FB_1_]non Bt {[(1 – BUF) – DUF] + (DUF × DCF)]}. Bt = *Bacillus thuringiensis*.

2 Source: Kansas State University Feed Intake Curve Calculator (FICC).

3 Source: USDA, 2010. Adoption of genetically engineered crops in the US: corn varieties.

4 *p* and *q* = beta generalized distribution shape parameters.

5 Source: [8].

6 Data modified from the Kansas State University swine nutritional guide. Grower-Finishing pig recommendations [9]. Corn was determined by the appropriate TCIF on the basis of body weight.

7 Cumulative distribution function (CDF).

8 Corn source derived from distiller’s dried grains with solubles (DDGS) is estimated to increase fumonisin B_1_ concentrations by a magnitude of 3.

**Table 2.** Body weight estimates by weekly intervals during grower-finishing phase production as determined from the Kansas State growth and feed intake curve calculator (FICC)^1^ and partitioned timeframes corresponding to total corn intake fraction (TCIF)^2^.

**Week**	**Weight, kg**	**Week**	**Weight, Kg**	**Portioned Weekly Timeframes**	**TCIF^2^**
1	27.2	11	85.5	Weeks 1 and 2	0.685
2	32.4	12	91.5	Weeks 3, 4, and 5	0.734
3	37.8	13	97.3	Weeks 6, 7, and 8	0.783
4	43.7	14	103.1	Weeks 9, 10, and 11	0.820
5	49.2	15	108.6	Weeks 12, 13, and 14	0.844
6	55.1	16	113.9	Weeks 15, 16, 17, 18, 19 and 20	0.864
7	61.1	17	118.9		
8	67.2	18	123.7		
9	73.3	19	128.2		
10	79.4	20	132.4		

1 FICC [7].

2 Data modified from the Kansas State University swine nutritional guide [9].

**Table 3.** Determination of total corn intake fraction (TCIF) in grower-finisher pig diets based on bodyweight^1^.

**Weight Ranges, kg**	**TCIF**
22.7 to 33.6	0.685
34.0 to 54.0	0.734
54.4 to 72.1	0.783
72.6 to 88.0	0.820
88.5 to 104.0	0.844
>104.3	0.864

1 Data modified from the Kansas State University swine nutritional guide [9].

**Table 4.** Percentage of insect-resistant *Bacillucs thuringiensis* (Bt) and stacked gene varieties (Bt plus herbicide resistance) in US 2010 corn varieties used to estimate Bt use fractions (BUF) in grower-finisher pig diets^1^ [4].

**State**	**% Insect-resistant Bt only**	**% Stacked genes varities**	**% Insect-resistant Bt only + % Stacked Gene Varieties**	**Fraction of insect-resistant Bt only + stacked gene varieties**
Illinois	15	52	67	0.67
Indiana	7	56	63	0.63
Iowa	15	61	76	0.76
Kansas	22	40	62	0.62
Michigan	11	44	55	0.55
Minnesota	18	46	64	0.64
Missouri	15	45	60	0.60
Nebraska	22	45	67	0.67
North Dakota	22	37	59	0.59
Ohio	13	36	49	0.49
South Dakota	6	60	66	0.66
Texas	18	40	58	0.58
Wisconsin	13	38	51	0.51
			Generalized β parameters^2^	
			Mean = μ	0.61
			Mode = *c*	0.67
			Maximum = *b*	0.76
			Minimum = *a*	0.49
			p = α1	0.67
			q = α1	0.83

1 USDA (2010), National Agriculture Statistics Service (NASS).

2 *p* and *q* = shape parameters.

**Table 5.** Deterministic and semi-stochastic predictions of grower-finishing pig exposure to fumonisin B_1_ (FB_1_) in diets.

**Feeding Scenarios^1^**	**Deterministic exposures mg FB_1_/kg diet**	**Semi-stochastic exposures mg of FB_1_/kg of diet**		
		**Median**	**Mean**	**90th**
**Scenario 1: Blended Diet^2^**	2.86	3.46	3.50	5.08
**Scenario 2: Bt grain & Bt DDGS**	2.32	2.25	2.40	4.01
**Scenario 3: non-Bt grain & non-Bt DDGS**	4.69	4.88	5.08	7.87
**Scenario 4: Bt & non-Bt grain**	2.09	2.13	2.19	3.20
**Scenario 5: Bt grain**	1.68	1.43	1.50	2.52
**Scenario 6: non-Bt grain**	3.40	3.02	3.11	4.97

1 Corn and corn derived component distiller dried grains with solubles (DDGS) in diet.

2 Includes a blend of Bt grain, non-Bt grain, Bt DDGS and non-Bt DDGS.
